# Element analysis applied to investigate acute kidney injury induced by red yeast rice supplement

**DOI:** 10.1007/s00795-024-00411-1

**Published:** 2024-11-13

**Authors:** Makoto Abe, Tadayuki Ogawa, Nobuyuki Magome, Yuko Ono, Akihiro Tojo

**Affiliations:** 1 Department of Nephrology and Hypertension, Blood Purification Center, Dokkyo Medical University, 880 Kitakobayashi, Mibu, Tochigi 321-0293 Japan; 2 Comprehensive Research Facilities for Advanced Medical Science, Dokkyo Medical University, Tochigi, Japan; 3 Department of Fundamental Education, Dokkyo Medical University, Tochigi, Japan; 4 Department of Diagnostic Pathology, Dokkyo Medical University, Tochigi, Japan

**Keywords:** Fanconi syndrome, Acute tubular necrosis, Functional food, Nanoparticle, Silica, Cholestehelp

## Abstract

Silica nanoparticles are used in functional foods and tablets to increase drug stability and delivery. We investigated a patient with acute kidney injury with Fanconi syndrome after taking functional food tablets made from red yeast rice using low-vacuum scanning electron microscopy (LVSEM) with an element analysis system. Kidney biopsy revealed proximal tubular necrosis and vacuolization with 10–20 nm black granules, which were similar to the silica nanoparticles found in the functional food tablets and urinary samples, as determined via LVSEM with element analysis. Reabsorbed silica nanoparticles induce oxidative stress in the kidney. Element analysis by LVSEM is useful to investigate a possible cause of acute tubular necrosis in patients with Fanconi syndrome.

**Supplementary Information:**

The online version contains supplementary material available at 10.1007/s00795-024-00411-1.

## Introduction

In Japan, functional food tablets made from red yeast rice (©Cholestehelp, CP, Kobayashi Pharmaceutical Co., Ltd., Osaka Japan) have become a social issue since some people have been diagnosed with acute kidney injury after consuming CP tablets [1–4]. It was found that some of the lots of CP tablets were contaminated with puberulic acid, which is produced by blue mold, but the mechanism by which puberulic acid-induced renal dysfunction is still unknown. Silica, which is considered an essential trace element [5], is found in many functional foods and tables. We analyzed a patient with acute kidney injury with Fanconi syndrome that was taking CP tablets and identified nanoparticles, including silica oxide, in the urine and kidney tissue samples via element analysis using low-vacuum scanning electron microscopy (LVSEM).

## Methods

### Ethics approval and consent to participate

Renal biopsy and sample analysis were performed with the approval of the clinical research ethics committee of Dokkyo Medical University (R-71-3 J).

### Transmission electron microscopy and LVSEM observation

Transmission electron microscopy of the renal biopsy sample and samples of the powdered Cholestehelp (CP) tablet dissolved in water was performed, and the samples were fixed with 2.5% glutaraldehyde, followed by the post-fixation with 1% osmium tetroxide and embedding in epoxy resin. Ultrathin sections were stained with uranyl acetate and lead citrate and observed under a transmission electron microscope (HT7800, Hitachi High-Tech Co., Tokyo, Japan). Urinary sediments from CP patient and patients with IgA nephropathy were directly observed via LVSEM (TM4000Plus, Hitachi High-Tech Co., Tokyo, Japan) with an acceleration voltage of 10 kV with mode 3 [6].

### Elemental analysis of kidney and urinary crystals and Si distribution

Elemental analysis of the urinary samples from the patients with CP-induced Fanconi syndrome and IgA nephropathy as well as samples of the powdered CP tablet dissolved in water was performed using a TM3030 low-vacuum scanning electron microscope (Hitachi, Tokyo, Japan) equipped with an energy dispersive X-ray spectroscopy (EDS) system, a Quantax 70EDS attachment (Bruker, Madison, WI, USA). The urinary sample and the supernatant of the powdered CP tablet were added dropwise onto a conductive tape and dried; images were taken at × 2000 magnification and 15.0 kV accelerating voltage. Quantax 70 software was used to obtain the elemental compositions from the EDS spectra [7].

### Immunohistochemistry for CD10, a marker of proximal tubules

Paraffin-embedded sections (2 µm-thick) were dewaxed, and immunostaining was performed with the Leica Autoimmune Stain System Bond III (Leica Microsystems, Tokyo, Japan) using antibodies against CD10 (Bond Ready-to-use primary antibody; Novo, Newcastle, UK) and an HRP-conjugated secondary antibody.

### Urinary protein fraction

For the analysis of the urinary protein fraction, 2.5, 5, or 7.5 *μ*L of urine was added to 2.5 *μ*L of sample buffer and diluted with distilled water to a final volume of 10 *μ*L, and samples were applied to a 4–10% gel (Daiichi Pure Chemicals, Tokyo, Japan) and separated by sodium dodecyl sulfate‒polyacrylamide gel electrophoresis (SDS‒PAGE). After electrophoresis, the gels were stained with Coomassie blue [8].

### Mitochondrial ROS production

Mitochondrial production of ROS was detected via a fluorometric mitochondrial superoxide assay using a MitoROS 520 kit (AATBioquest Inc., Cosmo Bio Co., Tokyo, Japan). The cryosections were incubated with MitoROS 520 in assay buffer for 1 h at 37 °C and observed under a fluorescence microscope (Keyence BZ-9000, Keyence, Osaka, Japan) with an excitation wavelength of 540 nm and an emission wavelength of 590 nm.

### Liquid chromatography–mass spectrometry (LC‒MS/MS)

Powdered CP tablets were dissolved in methanol, and 2 mL of the supernatant was concentrated to 50 μL and subjected to liquid chromatography. Because a peak was observed in the 5-min fraction of the tetraethoxysilane (TEOS) standard solution (Fujifilm Wako, Osaka, Japan), the 5-min fraction of the sample was collected and analyzed via LC‒MS/MS (SCIEX QTRAP 5500, Framing, Massachusetts, USA). Characteristic peaks were compared to those of TEOS standard solutions.

## Results

### Patient

A 50-year-old man was admitted to our hospital due to complaints of appetite loss and body weakness. Before his symptoms developed, he had been consuming CP tablets for 7 months to help lower his low-density lipoprotein cholesterol level. He had normal renal function before taking any medications. However, the patient was diagnosed with acute kidney injury with Fanconi syndrome on the basis of the following data: serum creatinine (Cr), 3.41 mg/dL; estimated glomerular filtration rate (eGFR), 16.5 mL/min/1.73 m^2^; high urinary protein levels; high urinary glucose levels; amino aciduria; hypokalemia; hypophosphatemia; and hypouricemia (Table 1). Four months after cessation of CP intake, his proteinuria disappeared, but his renal function, although it had partially improved, did not completely recover.

**Table 1:** Laboratory data at renal biopsy and 4 months later

Blood test	At biopsy	4 months later	Urine test	At biopsy	4 months later
Alb g/dL	4.8	4.7	specific gravity	1.012	1.005
UN mg/dL	33.3	19.8	pH	6.0	6.5
Cr mg/dL	3.99	1.88	Protein g/gCr	2.6	0.15
eGFR ml/min/1.73m^2^	13.9	31.6	Glucose mg/dL	2000	−
Na mEq/L	135	137	Red blood cells/HPF	Rare	Rare
K mEq/L	2.8	4.3	White blood cells/HPF	Rare	Rare
Cl mEq/L	106	105	Cast/HPF	Rare	Rare
Ca mg/dL	8.8	9.9	FE_Na_ %	1.35	1.09
IP mg/dL	2.4	3.4	FE_K_ %	48.8	26.9
Mg mg/dL	2.4	2.2	FE_UA_ %	84	13.1
UA mg/dL	1.8	5.6	%TRP	20.4	73.1
Glu mg/dL	87	92	NAG U/L	13	2.7
pH	7.204	7.358	β_2_-MG µg/L	34,200	1630
HCO_3_^−^ mEq/L	9.6	25.3	SI	0.046	nd
			Aminoaciduria	+	nd

*HPF* high-power field, *FE*_*Na*_ fractional clearance of sodium, *FE*_*K*_ fractional clearance of potassium, *FE*_*UA*_ fractional clearance of uric acid, *%TRP* percent reabsorption of phosphate, *NAG* N-acetyl β-glucosamidase, *SI* selective index, *nd* not done

### Renal biopsy

**Fig. 1:**
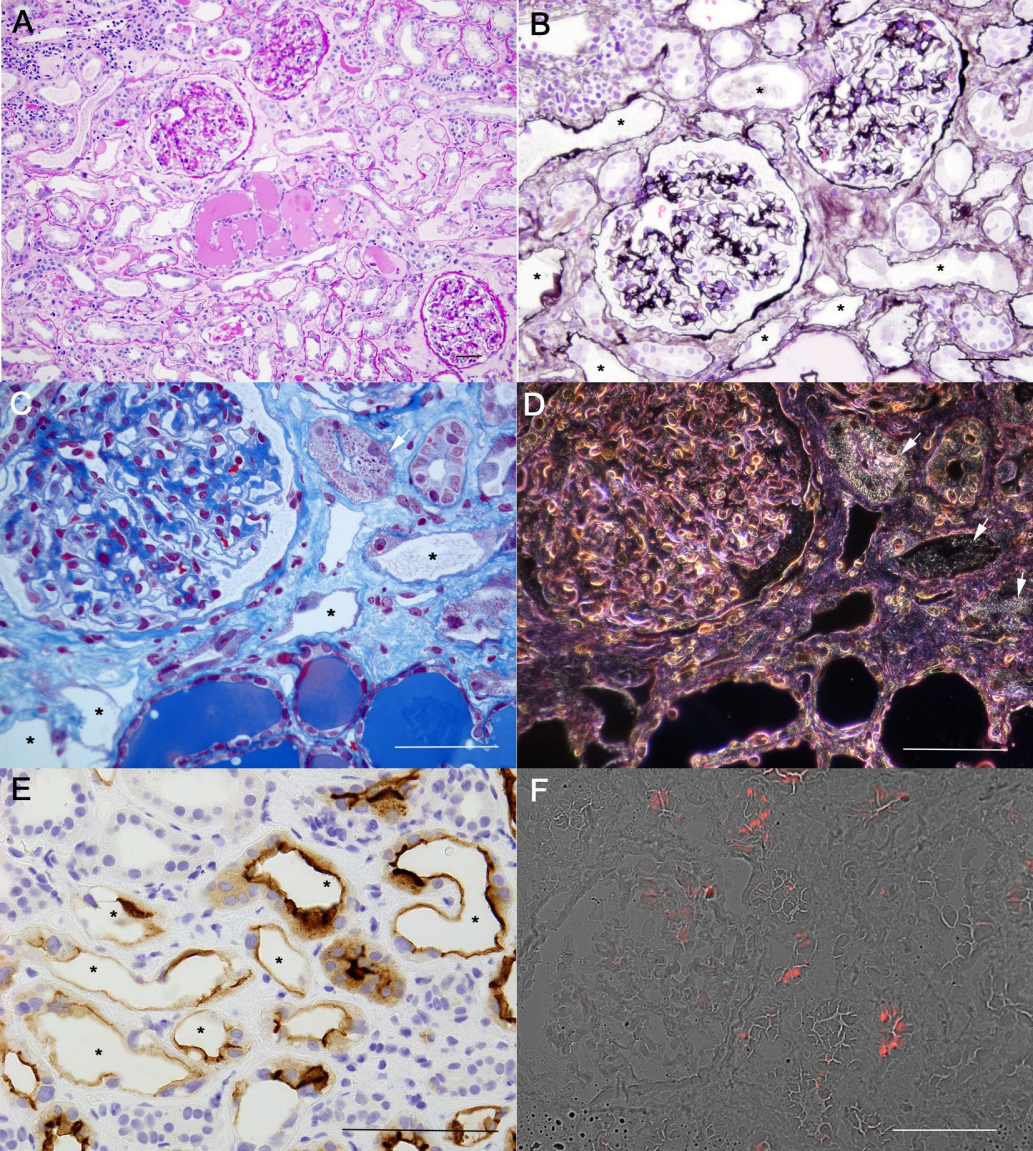
Kidney biopsy findings. PAS staining (**A**), PAM staining (**B**), AZAN staining (**C**) and dark-field microscopy (**D**) show granular particles (arrows) and immunostaining of the proximal tubular marker CD10 (**E**), with proximal tubular cells showing degeneration, detachment or flattened morphology (stars). Mitochondrial production of reactive oxygen species (**F).** Scale bars = 50 μm

**Fig. 2:**
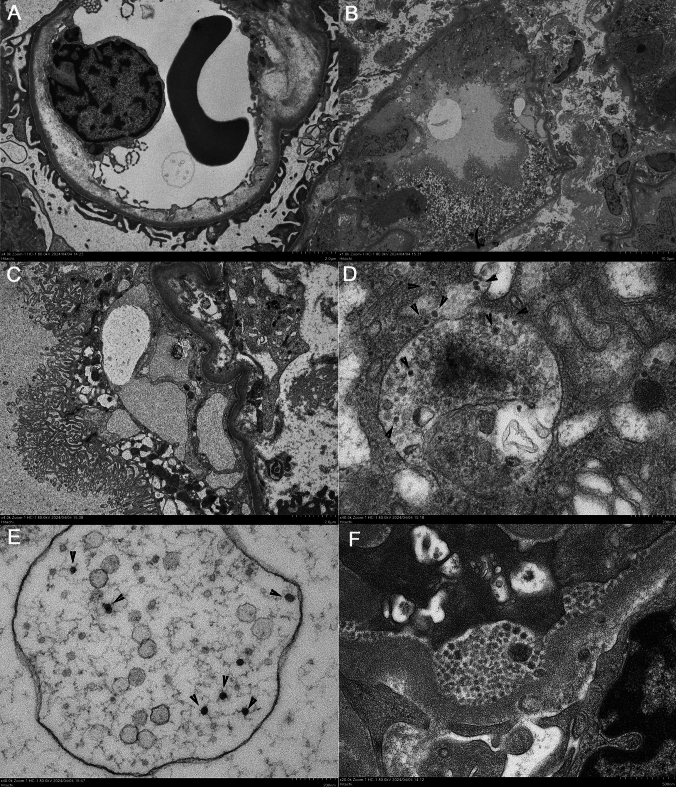
Transmission electron microscopy image of a renal biopsy sample showing the glomerulus (**A**, **F**) and proximal tubules (**B**–**E**). Arrow heads indicate nanoparticles. Magnification at × 1000 (**B**), × 4000 (**A**, **C**), × 20,000 (**F**), and × 40,000 (**D**, **E**)

Renal biopsy revealed acute tubular necrosis (ATN) with tubular cells showing degeneration, detachment or flattened morphology (Fig. 1A–C), and immunostaining for CD10 indicated that the damaged tubules were proximal tubules (Fig. 1E). Azan staining revealed white birefringent granular materials within the cells and lumen of the proximal tubules under dark-field microscopy (Fig. 1C, D). The glomeruli were normal without any significant immunofluorescence staining findings. Electron microscopy revealed subendothelial space expansion, indicating glomerular endothelial damage (Fig. 2A), and vacuolar degeneration of the proximal tubule, with mitochondrial damage (Fig. 2B, C). Interestingly, 10–20 nm black granules and 100 nm vesicles were identified within the lysosomes and tubular lumen (Fig. 2D, E) as well as under the glomerular basement membrane (Fig. 2F). These nanoparticles may contribute to proximal tubular damage and increase mitochondrial ROS production (Fig. 1F), which can lead to ATN.

### Urinary protein analysis

**Fig. 3:**
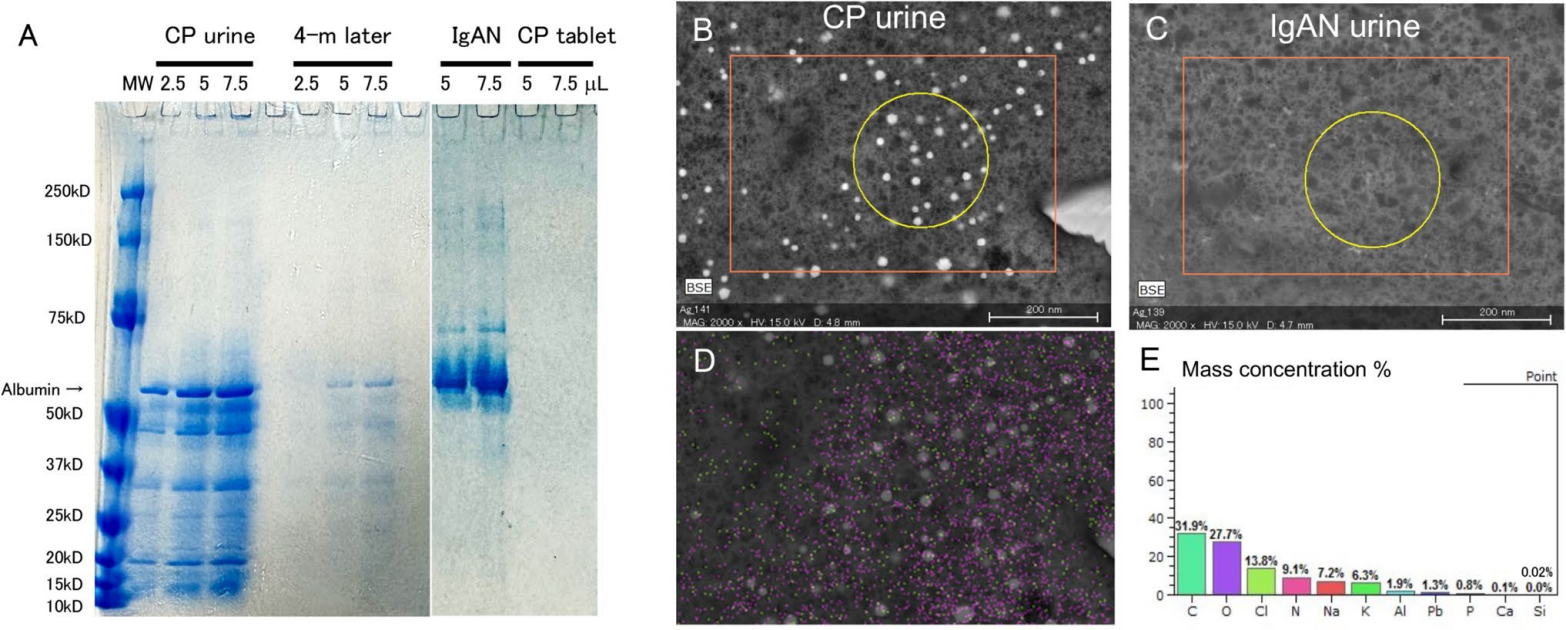
Urinary analysis with SDS‒polyacrylamide gel electrophoresis (PAGE) (**A**) and elemental analysis (**B**‒**E**). SDS‒PAGE of urinary proteins from patients with CP-induced Fanconi syndrome or IgA nephropathy at biopsy and 4 months later as well as the supernatant of powdered CP tablets dissolved in water (**A**). LVSEM images of the urine samples of patients with CP (**B**) and IgA nephropathy (**C**), elemental analysis of the urine of CP patient indicating Si with green dots and O with purple dots (**D**), and their mass concentrations (**E**)

The urinary protein fraction of patients with CP-induced Fanconi syndrome showed mainly low-molecular-weight proteins (LMWPs) less than 40 kDa, which were rarely observed in the urine 4 months after cessation of CP or in the urine of patients with IgA nephropathy. (Fig. 3A).

The urinary sample of the CP patient had 20–100 nm vesicles containing silicon (Si), whereas no vesicles were observed in the urinary sample of a patient with IgA nephropathy (Fig. 3B–E).

### Identification of silica nanoparticles in the Cholestehelp tablets

**Fig. 4:**
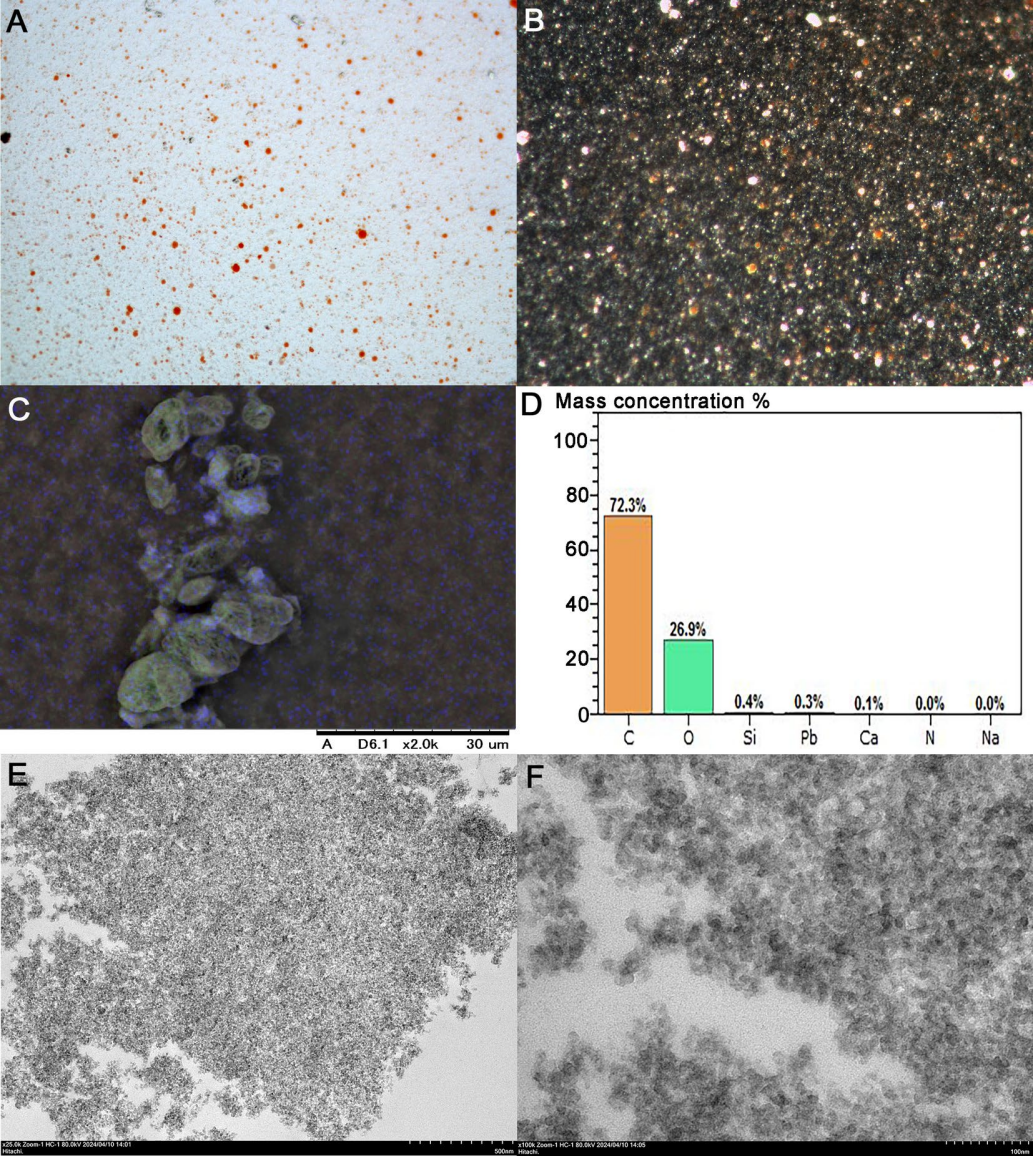
Analysis of powdered CP tablets. Light microscopy image (**A**) with dark-field illumination (**B**) × 400. Elemental analysis with LVSEM-EDS indicated blue dots for Si and green dots for O (**C**, **D**). Transmission electron micrographs of powdered CP tablets with particle sizes of 10–20 nm (**E**, **F**)

The powdered CP tablet was poorly dissolved in water, and in addition to the orange-colored vesicles with a size of 100–200 nm, smaller fine particles that emitted white polarized light were observed in the supernatant (Fig. 4A, B). Elemental analysis via low-vacuum scanning electron microscopy (LVSEM) equipped with energy dispersive X-ray spectroscopy (EDS) revealed that the lumpy CP powder contained unevenly distributed Si in addition to C and O (Fig. 4C, D). The granule size of the CP tablets was 10–20 nm (Fig. 4E, F), which was approximately the same size as the black granules observed in the proximal tubules.

## Discussion

In this study, nanoparticles were identified in the proximal tubules of an AKI patient with Fanconi syndrome after the consumption of silica nanoparticle-containing Cholestehelp tablets, along with vacuolar changes and cell detachment. Thus, we propose a novel mechanism of renal damage caused by the functional food red yeast rice rather than by the presence of puberulic acid.

**Fig. 5:**
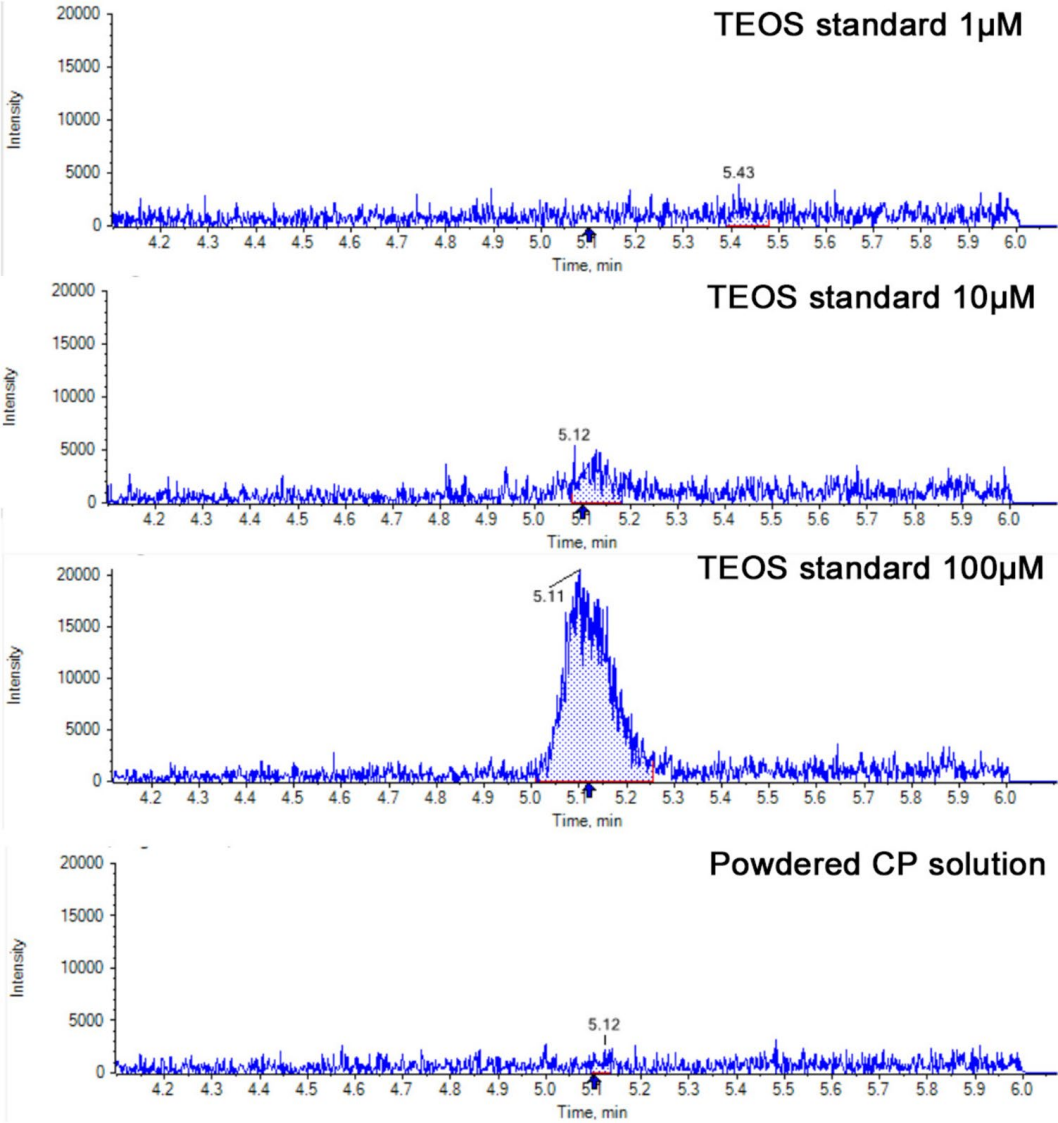
LC‒MS/MS analysis of the tetraethoxysilane (TEOS) standard and powdered CP solutions. TEOS was not detected in the CP table. The TEOS concentration in the powdered CP solution may be lower than the sensitivity of the LC‒MS/MS system

Silica, a safe additive used as a suitable delivery vehicle, has been shown not to cause damage to organs [9]. However, the ability of silica nanoparticles to enter the body has been recognized as toxic [10, 11], as they impair normal kidney and liver functions via oxidative stress in animal studies [12]. Silica nanoparticles are produced from flammable liquid tetraethoxysilane [Si(OC_2_H_5_)_4_, TEOS] [13], and the particle size changes from 10 to 200 nm according to the reaction temperature and time [14]. TEOS is known to cause ATN [15, 16]. The residual TEOS during silica nanoparticle production could contaminate many CP tablets, which may have caused ATN. However, TEOS was not detected in the CP tablets consumed by the patient (Fig. 5). The sensitivity of LC‒MS/MS was not high enough to distinguish TEOS concentrations below 10 mM, so the possibility of TEOS contamination cannot be completely ruled out. No protein bands were observed in the extracted CP tablets (Fig. 3A). Therefore, the protein components derived from the red yeast rice were not present in the patient’s urine. Spherical granules, which are thought to be silica nanoparticles, were found in the patient’s urine but not in the urine of IgA nephropathy patients (Fig. 3).

The reported cases of AKI due to the ingestion of red yeast rice CP are summarized in Table 2. Fanconi syndrome was observed in all patients, and proteinuria was completely ameliorated after CP tablet ingestion was discontinued, suggesting that proximal tubular damage can be reversed simply by discontinuing CP tablet ingestion. Although the serum creatinine levels were partially restored, renal dysfunction persisted in three patients. In one patient with short-term CP intake [3] and one patient with mild creatinine elevation one year after CP intake [4], renal function appeared to be completely restored with steroid therapy. However, since no obvious lymphocytic infiltration or immune abnormalities were observed, further investigation is needed to determine whether steroid therapy is effective in treating proximal tubular damage caused by CP tablet intake. Interestingly, Kawai et al. [4] reported the presence of virus-like electron-dense particles in the proximal tubules, which may be consistent with our observations of silica nanoparticles. We have previously reported animal studies using Eucommia tea extract containing silica nanoparticles (Kobayashi Pharmaceutical Co., Ltd., Osaka) [17], and using kidney samples, we newly discovered Si-containing nanoparticles within the lysosomes of proximal tubules in rats administered Eucommia tea extract (Supplementary Fig. 1). Further studies are needed to investigate the role of silica nanoparticles as a cause of ATN.

**Table 2:** Reported cases of acute kidney injury due to Cholestehelp (CP)

Patients	Age sex	CP intake periods	Cr mg/dL	K mEq/L	P mg/dL	UA mg/dL	HCO_3_^−^ mEq/L	UP g/gCr	U-RBC	U-glu	NAG IU/L	U-β2-MG μg/L	Treatment	Final Cr, UP
Miyazaki [2]	47 F	9 months	4.26	3.6	3.7	2.5	n.d	1.38	5–9/HPF	4 +	16.6	109,677	PSL40 mg	Cr1.72, UP (-)
Maiguma [3]	58 F	6 weeks	2.70	4.3	2.9	1.7	n.d	1.06	negative	3 +	71.7	126,473	PSL20 mg	Cr0.65, UP (-)
Kawai [4]	56 F	1 year	1.39	3.2	1.4	1.4	15.6	2.34	1–4/HPF	3 +	26.7	82,520	PSL30 mg	Cr0.88, UP 0.16
Oda [1]	62 M	1.5 year	1.43	2.6	1.1	1.0	20.3	1.25	1–4/HPF	3 +	40.0	41,475	Potassium 4-8 mEq/day Phosphorus 300 mg	Cr1.21, UP (-)
Present case	50 s M	7 months	3.99	2.8	2.4	1.8	9.6	2.60	1–3/LPF	4 +	13.0	34,200	NaHCO_3_ 1 g	Cr1.88, UP 0.15

*UP* urinary protein, *NAG* N-acetyl β-glucosamidase, *β2-MG* β2-microglobulin, *HPF* high-power field, *LPF* low-power field

In conclusion, element analysis using LVSEM is useful to investigate the possible cause of acute tubular necrosis with Fanconi syndrome by red yeast rice supplement.

## Supplementary Information

Below is the link to the electronic supplementary material.

Supplementary file1 (DOCX 19211 KB)

## Data Availability

The data generated and analyzed during the current study are available from the corresponding author on reasonable request.
