# Supplementary material for: Element analysis applied to investigate acute kidney injury induced by red yeast rice supplement
Source: Med Mol Morphol. 2024 Nov 13;58(1):53–61. doi: 10.1007/s00795-024-00411-1 (PMC11829840; doi:10.1007/s00795-024-00411-1)
Supplement: Supplementary file 1 — Supplementary file1 (DOCX 19211 KB) [file 795_2024_411_MOESM1_ESM.docx]

**Supplementary file**

We have previously reported effect of Eucommia ulmoides (Tochu) on blood pressure and renal hemodynamics [1]. In this experiments male Dahl salt sensitive rats received 1% salt drinking water (DSHS rats) were with 500 mg/kg body weight of Tochu tea extract (Kobayashi Pharmaceutical Inc, Osaka, Japan) in drinking water, which also include SiO2 as same as Cholestehelp. The rats treated with Tochu tea extract (DSHS+T) showed accumulated granules in the proximal tubules (Supplementary Figure 1 A, C, E) compared with those in DSHS rats (Supplementary Figure 1 B, D, F). Element analysis showed the nanoparticles included Si were identified more in DSHS+T rat compared to DSHS rat (Supplementary Figure 1 G, H). These data indicate that silica-containing nanoparticles contained in the functional food Eucommia tea extract can be reabsorbed in the proximal tubules of hypertensive rats with proteinuria. Therefore, the silica nanoparticles contained in Cholestehelp may also be reabsorbed in the proximal tubules in some patients with proteinuria.
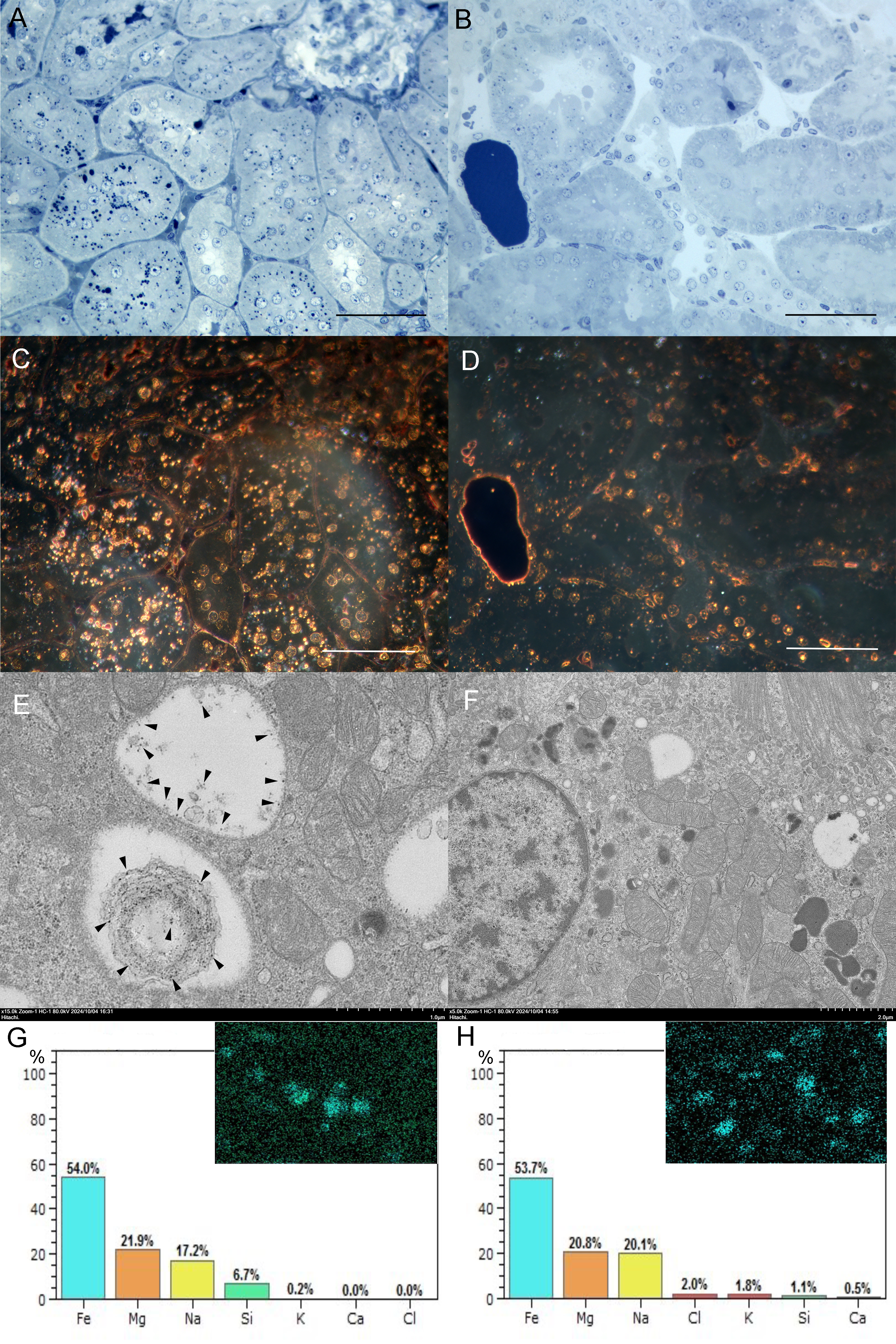


Supplementary Figure 1. Toluidine blue staining of kidney section of DSHS rat treated with Tochu tea extract with silica (A, C, E) and control DSHS rat (B, D, F). Dark-field microscopic observation of the kidney section (C, D). The bars indicate 50-μm. Electron microscopy demonstrated nanoparticles in the lysosomes of DSHS treated with Tochu tea extract (E) but not in DSHS rat (F). Element analysis by LVSEM-EDS showing Si (green dots) and Fe (blue dots) and their % weight concentration(G, H).

References

1. Ishimitsu A, Tojo A, Satonaka H, Ishimitsu T (2021) Eucommia ulmoides (Tochu) and its extract geniposidic acid reduced blood pressure and improved renal hemodynamics. Biomed Pharmacother 141:111901. doi:10.1016/j.biopha.2021.111901
